# Characterization of pectin methylesterase gene family and its possible role in juice sac granulation in navel orange (*Citrus sinensis* Osbeck)

**DOI:** 10.1186/s12864-022-08411-0

**Published:** 2022-03-07

**Authors:** Zixuan Li, Liming Wu, Ce Wang, Yue Wang, Ligang He, Zhijing Wang, Xiaofang Ma, Fuxi Bai, Guizhi Feng, Jihong Liu, Yingchun Jiang, Fang Song

**Affiliations:** 1 Institute of Fruit and Tea, Hubei Academy of Agricultural Sciences, Wuhan, 430064 PR China; 2 College of Horticulture and Forestry Sciences, Huazhong Agricultural University, Wuhan, 430070 PR China

**Keywords:** *Citrus sinensis*, Pectin methylesterases (PMEs), Juice sac granulation, Transcription factor

## Abstract

**Background:**

Citrus is one of the most important fresh fruit crops worldwide. Juice sac granulation is a physiological disorder, which leads to a reduction in soluble solid concentration, total sugar, and titratable acidity of citrus fruits. Pectin methylesterase (PME) catalyzes the de-methylesterification of homogalacturonans and plays crucial roles in cell wall modification during plant development and fruit ripening. Although *PME* family has been well investigated in various model plants, little is known regarding the evolutionary property and biological function of *PME* family genes in citrus.

**Results:**

In this study, 53 non-redundant *PME* genes were identified from *Citrus sinensis* genome, and these *PME* genes were divided into four clades based on the phylogenetic relationship. Subsequently, bioinformatics analyses of gene structure, conserved domain, chromosome localization, gene duplication, and collinearity were performed on *CsPME* genes, providing important clues for further research on the functions of *CsPME* genes. The expression profiles of *CsPME* genes in response to juice sac granulation and low-temperature stress revealed that *CsPME g*enes were involved in the low temperature-induced juice sac granulation in navel orange fruits. Subcellular localization analysis suggested that *CsPME* genes were localized on the apoplast, endoplasmic reticulum, plasma membrane, and vacuole membrane. Moreover, yeast one-hybrid screening and dual luciferase activity assay revealed that the transcription factor *CsRVE1* directly bound to the promoter of *CsPME3* and activated its activity.

**Conclusion:**

In summary, this study conducts a comprehensive analysis of the *PME* gene family in citrus, and provides a novel insight into the biological functions and regulation patterns of *CsPME* genes during juice sac granulation of citrus.

**Supplementary Information:**

The online version contains supplementary material available at 10.1186/s12864-022-08411-0.

## Background

The plant cells are surrounded by a rigid extracellular structure called cell wall which is composed of polysaccharides (cellulose, hemicelluloses, and pectin), phenolics, and cell wall proteins [1, 2]. Pectin, the major constituent of the plant cell wall, is a hetero-polysaccharides containing homogalacturonan (HG), rhamnogalacturonan-I (RG-I), rhamnogalacturonan II (RG-II), and xylogalacturonan (XGA) components [3, 4]. HG, a linear homopolymer of a-1,4-linked galacturonic acid, is the dominant form of pectic polysaccharide, accounting for ~ 65% of total pectin in plant cells [5]. HG is synthesized in Golgi apparatus and delivered to the apoplast in a highly methylesterified state [6].

Pectin methylesterase (PME), also known as pectinesterase, catalyzes the removal of the methyl group from HG, thus generating methanol and negatively charged carboxyl groups in the de-methylesterification process [7]. The de-methylesterified HG is often susceptible to be degraded by pectin-degrading enzymes including polygalacturonases and pectate lyases, which may loosen the cell wall [8–10]. Alternatively, when the degradation process is blocked, de-methylesterified HG is able to cross-link with calcium ions to form a structure called “egg box”, which may rigidify the cell wall [11, 12]. The extent of HG de-methylesterification may be affected by PME inhibitors (PMEIs) which directly bind to the catalytic domain of PMEs to inhibit their enzyme activity [7, 11]. According to the protein structure, the PMEs fall two groups, namely, Type-I PMEs (with PME domain and an additional N-terminal pro-region similar to PMEI domain) and Type-II PMEs (with PME domain only) [13]. PME is widely present in higher plants. Genome-wide identification of *PME* gene family has been reported in many plant species. Specifically, 66 PME genes were identified from *Arabidopsis thaliana* [14], 110 from *Brassica rapa* [15], 80 from *Gossypium arboretum* [16], 43 from *Oryza sativa* [17], 105 from *Linum usitatissimum* [18], and 57 from *Lycopersicon esculentum* [19]. Previous studies have revealed that *PME* genes exert various functions in plants, such as fruit ripening [20, 21], fruit softening [22], pollen development and growth [23, 24], root hair formation, seed germination [25], as well as biotic and abiotic stress response [26–29].

Citrus, as one of the most important fresh fruit crops worldwide, is valued for its high levels of functional components, including volatiles [30], flavonoids [31, 32], carotenoids [33, 34], and polysaccharides [35]. However, the juice sac granulation is a serious physiological disorder that often occurs in the on-tree fruits of late-ripening citrus cultivars and postharvest fruits of normal citrus cultivars during storage [36, 37]. Granulation leads to a great reduction in soluble solid concentration, total sugar, and titratable acidity, thus decreasing the edible value and commercial value of citrus fruits [36, 38, 39]. Previous studies have suggested that pectin is significantly accumulated in granulated citrus fruits, and the expression profiles of *PME* genes are significantly changed during granulation, indicating a strong association of *PME* genes with citrus fruit granulation [37, 39]. However, the knowledge of *PME* family genes in citrus remains very limited.

In this study, a genome-wide analysis was performed to identify the whole *PME* family genes in *Citrus sinensis*. The phylogenetic relationship, gene structure, conserved domain, chromosomal localization, gene duplication events and collinear correlation of *CsPME* family genes were analyzed. Furthermore, the spatial and temporal expression patterns of *CsPME* genes and their expression profiles in response to juice sac granulation and low-temperature treatments were assessed. Moreover, the yeast one-hybrid screening and dual luciferase activity assay were performed to investigate the regulation network of *CsPME* genes during juice sac granulation. Our results extend the knowledge of citrus *PME* genes and provide an important basis for further research on their functions during juice sac granulation of citrus fruits.

## Results

### Genome-wide identification of *PME* family genes from* Citrus sinensis*

To identify *PME* family genes from *C. sinensis*, HMMER and BLASTP analyses of the sweet orange genome (*C. sinensis*, version 2) were performed [40]. The predicted CsPME proteins containing both PME and PMEI domains were designated as Type-1 PMEs, and the proteins containing only PME domain (but no PMEI domain) were designated as Type-2 PMEs. After verification with CDD and SMART, a total of 53 non-redundant CsPME proteins were identified from *C. sinensis* genome, including 29 Type-I and 25 Type-II (Supplementary Table S1). These 53 *CsPME* genes were named according to their physical location on the chromosomes. Basic information on *CsPME* genes, including their gene name, locus ID, number of amino acid (aa) residues, molecular weight (Da), isoelectric point (pI), grand average of hydropathicity (GRAVY), instability index (II), and gene position, was shown in Supplementary Table S2. The CsPME proteins varied from 144 to 632 aa in length with a predicted molecular weight ranging from 16,814.69 to 69,600.03 Da and a theoretical isoelectric point (pI) from 4.68 to 10.69.

### Phylogenetic analysis of *CsPME* genes

**Fig. 1:**
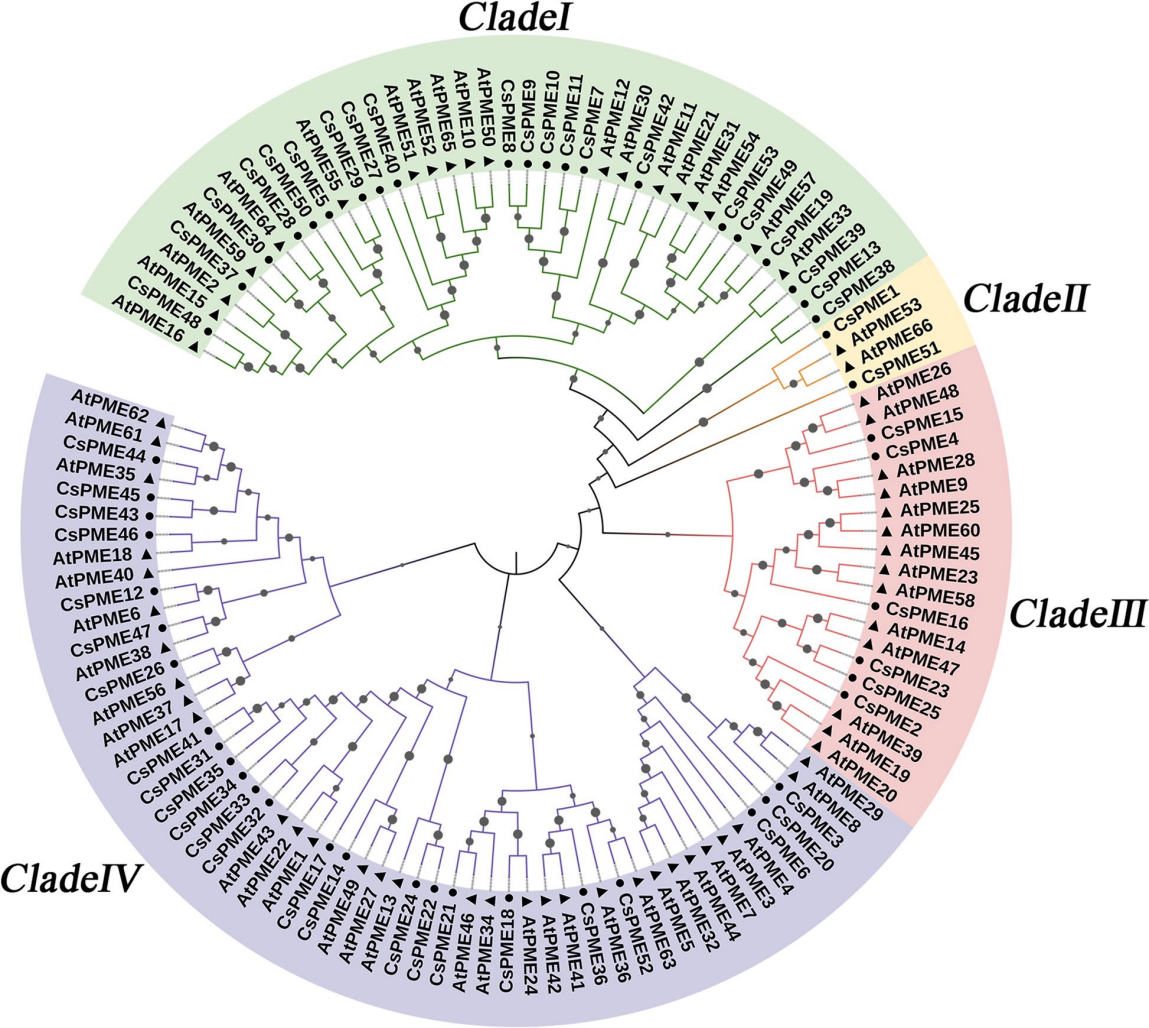
Phylogenetic relationship of *PME* genes between *Citrus sinensis* and *Arabidopsis thaliana*. The phylogenetic tree was constructed by MEGA X using the Maximum Likelihood Method (1000 bootstrap). The green, yellow, red, and purple backgrounds indicate the four subfamilies (Clade I, II, III, IV) of *PMEs* in *Citrus sinensis* (circle) and *Arabidopsis thaliana* (triangle). The probability of each branch was indicated with solid circles on the top of the branch, and the bigger solid circles indicated higher probabilities

To explore the phylogenetic relationship of *CsPMEs* between citrus and Arabidopsis, a phylogenetic tree was constructed with the amino acid sequences of 53 CsPMEs and 66 AtPMEs by using the Maximum Likelihood Method of MEGA X. As shown in Fig. 1, the *PME* family was clustered into 4 clades according to the previous definition of *AtPMEs* [14]. The Type-I *CsPMEs* (with a PMEI domain) were clustered into Clade II, Clade III, and Clade IV, whereas Type-II *CsPMEs* (without a PMEI domain) were mainly clustered into Clade I.

### Conserved motif and gene structure analyses of *CsPME* genes

**Fig. 2:**
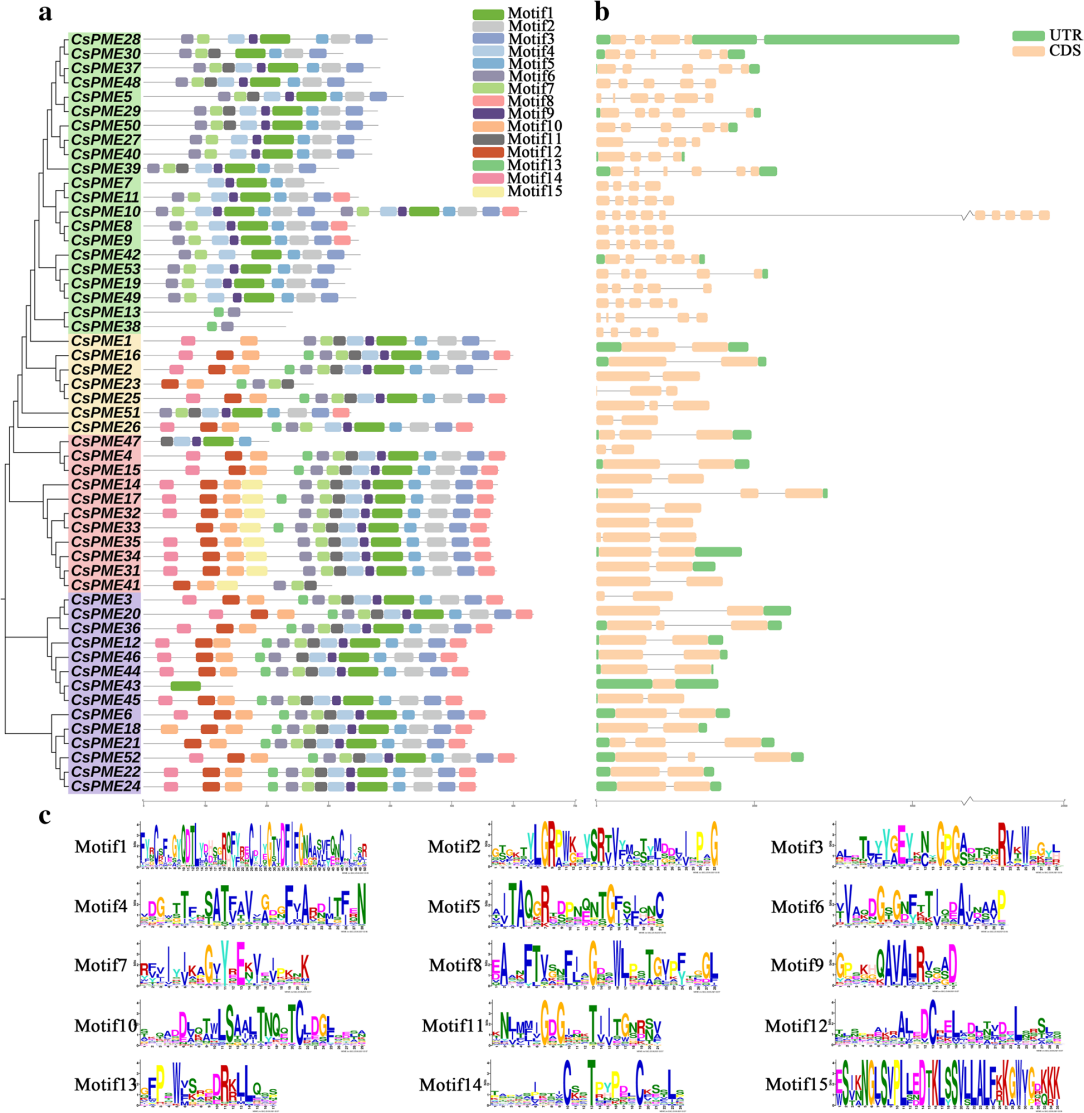
Conserved motif and gene structure analyses of *PME* genes in *Citrus sinensis*. **a** The conserved motif distribution of CsPME proteins. Each motif is marked with a number in a colored box (motifs 1 to 15). **b** Exon–intron structure of *CsPME* genes. Exons and introns are shown by green rectangles and thin lines. **c** The conserved amino acids of all the conserved motifs. The untranslated regions (UTRs) are indicated by carnation rectangles. Motif 1, 2, 3, 4, 5, 6, and 7 were pectinesterase activity domains, and motif 10 was pectinesterase inhibitor activity domain

The conserved motifs were analyzed based on the coding sequences of *CsPMEs*. As shown in Fig. 2a, a total of 15 conserved motifs were predicted in CsPME proteins using MEME website, and the conserved protein sequences were displayed in Fig. 2c, and the size of the motifs ranged from 15 (motif 9) to 50 aa (motif 1). Most *PME* members in the same clade shared similar conserved motifs. Based on the InterPro database, motif 1, 2, 3, 4, 5, 6, and 7 were annotated as pectinesterase activity domains. Motif 1, 2, 3, 4, 5, 6, and 7 were present in 49, 47, 46, 47, 48, 50, and 48 CsPME proteins, indicating that these pectinesterase-related motifs were highly conserved in CsPME proteins. Most interestingly, motif 10 was annotated to encode the PMEI domain, which was in line with the result that motif 10 was presented in 29 Type-I CsPME proteins.

To further characterize the gene structural diversity of *PME* family in sweet orange, the exon–intron organizations were analyzed based on the genome sequence of *CsPME* genes. The number of exons varied from 2 to 10. The gene structure analysis also revealed that *CsPME* genes in the same clade usually shared similar gene structures. As shown in Fig. 2b, the exon numbers of Type-II *CsPME* genes were relatively larger than that of Type-I *CsPME* genes, but their exon sizes were relatively smaller. The conservation of both conserved motif and gene structure of *CsPME* family members in the same clade strongly supported the reliability of the phylogenetic classification in Fig. 1.

### Chromosomal localization, gene duplication, and synteny analysis

**Fig. 3:**
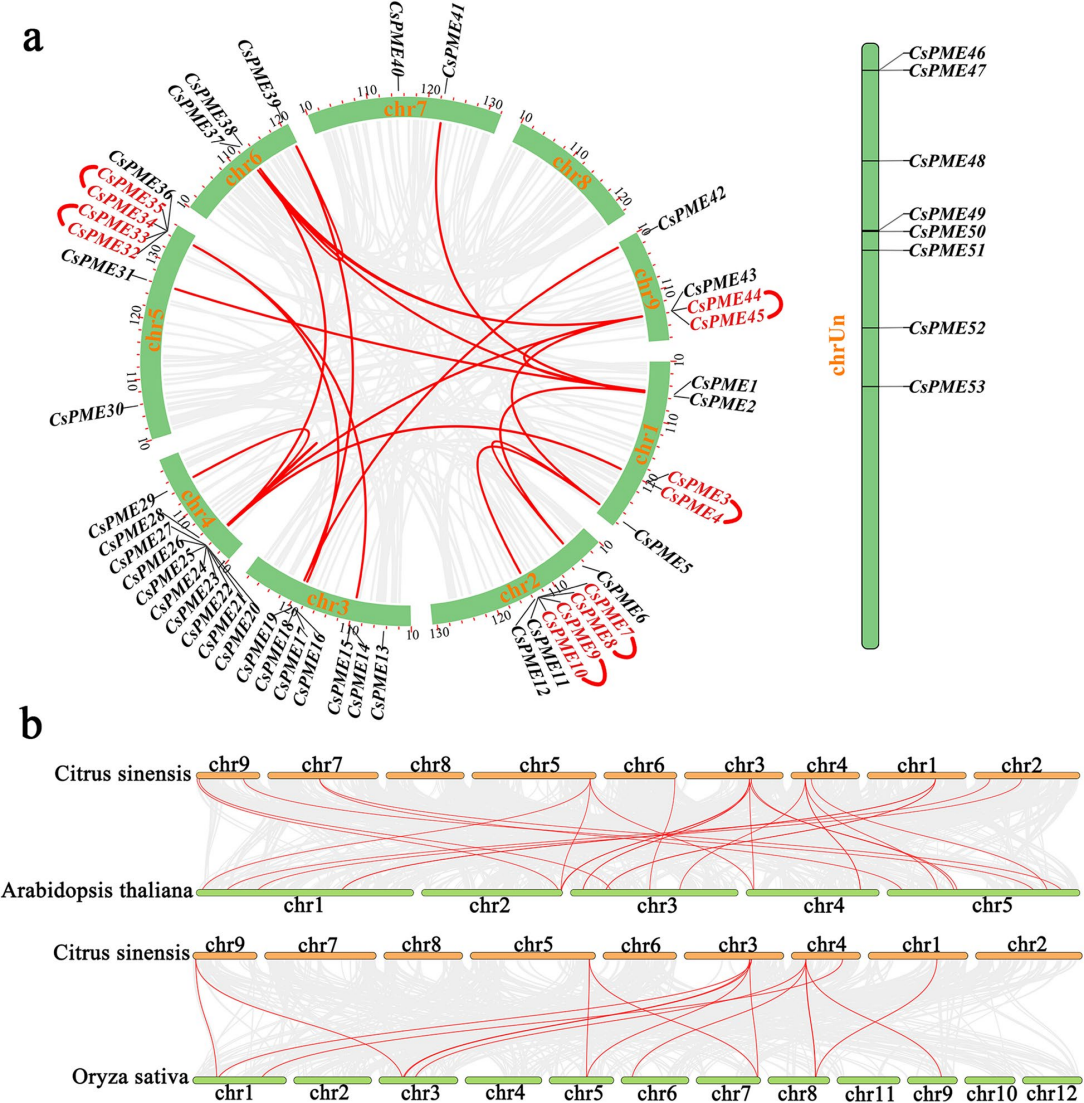
Chromosomal localization, gene duplication, and colinearity analyses of *CsPME* genes. **a** Chromosomal Localization and collinear relationship of *PME* genes within *Citrus sinensis*. The tandemly duplicated genes are indicated by red letters and segmentally duplicated genes are linked with red lines. **b** The collinearity of *PME* genes between *Citrus sinensis*, *Arabidopsis thaliana*, and *Oryza sativa*. The gray curved lines represent the collinear blocks in the whole genome of citrus and other plants. The red curved lines represent collinear *PME* gene pairs between *Citrus sinensis*, *Arabidopsis thaliana*, and *Oryza sativa*

Genome chromosomal localization analysis revealed that 53 *CsPME* genes were distributed on 8 chromosomes except Chromosome 8 (Fig. 3a). Chr 4 had the largest number (10) of *CsPME* genes, follow by Chr 2 (7) and Chr 5 (7). Additionally, 8 *CsPME* genes were failed to be localized on the total 9 chromosomes. Gene duplication provided raw materials for generating new genes and functions. The gene duplication analysis of *CsPMEs* revealed that 6 pairs of *CsPME* genes (corresponding to 12 *CsPME* genes) were identified as tandemly duplicated genes, and 17 pairs of *CsPME* genes (corresponding to 19 *CsPME* genes) were identified as segmentally duplicated genes (Fig. 3a). These results indicated that segmental duplication might contribute more to the expansion of *CsPME* genes compared to tandem duplication.

To further identify the orthologous genes of *CsPMEs* as well as their evolution relationship, two comparative syntenic relationship was established based on two representative species, *A. thaliana* (dicot) and *O. sativa* (monocot). As shown in Fig. 3b, a total of 27 orthologous *PME* gene pairs were identified between citrus and Arabidopsis, and 16 orthologous *PME* gene pairs were identified between citrus and rice. Interestingly, some orthologous *PME* gene pairs identified between citrus and rice were not detected between citrus and Arabidopsis, such as *CsPME22*/*OsPME21*, *CsPME25*/*OsPME29*, and *CsPME29*/*OsPME7*, suggesting that these orthologous gene pairs were generated after the divergence of dicotyledonous and monocotyledonous plants. Additionally, one citrus *PME* gene usually matched three or more Arabidopsis *PME* genes. For example, *CsPME3* was orthologous to *AtPME8*, *AtPME9*, *AtPME28*, and *AtPME29*, and *CsPME32* to *AtPME1*, *AtPME22*, and *AtPME43*, which implied that these genes in *A. thaliana* were paralogous gene pairs.

### Spatial and temporal expression patterns of *CsPME* genes in citrus fruits

**Fig. 4:**
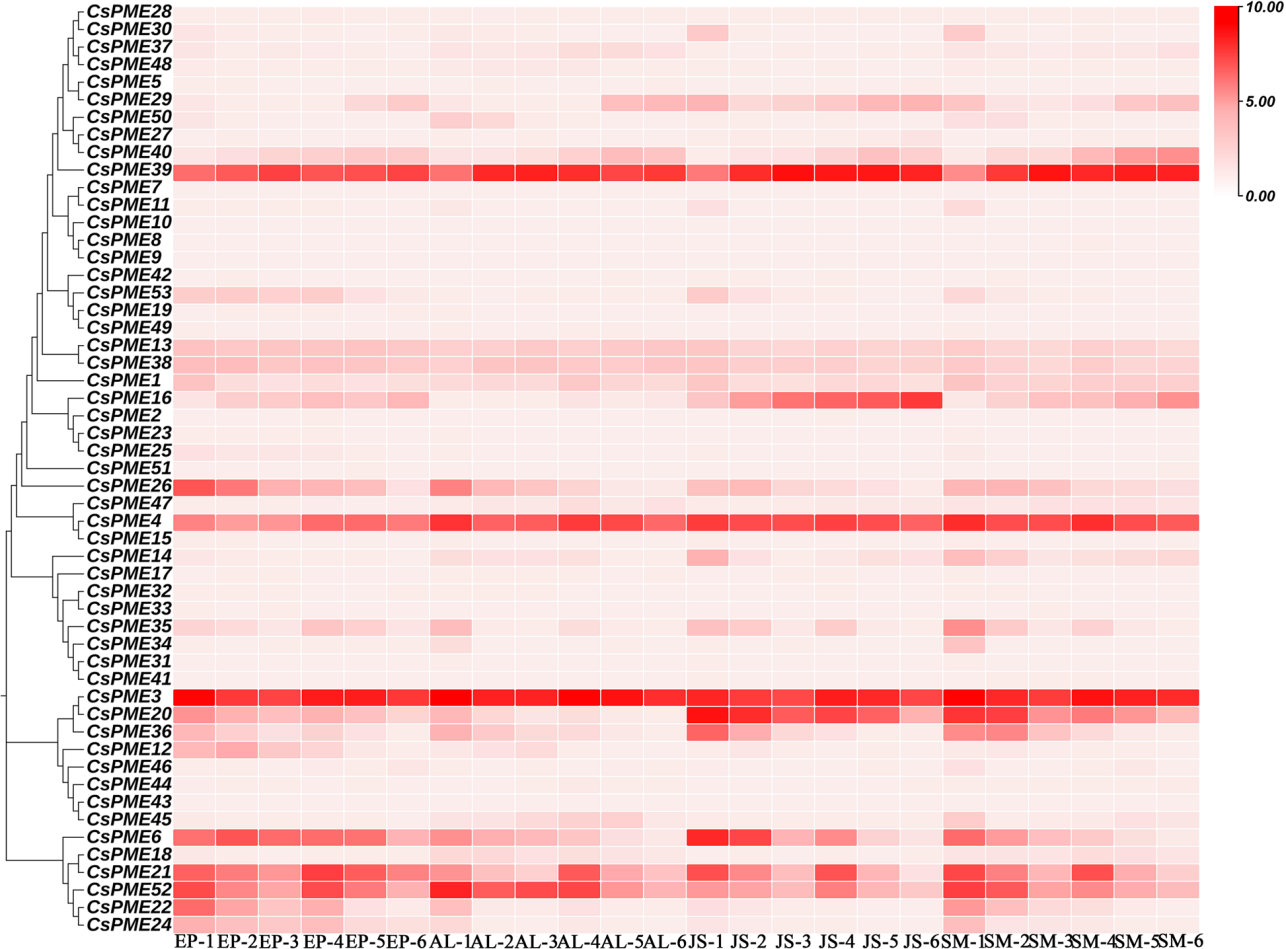
Spatial and temporal expression patterns of *CsPMEs* in 4 fruit tissues at 6 development stages in navel orange. Four fruit tissues including albedo (AL), epicarp (EP), juice sacs (JS), and segment membrane (SM), and six fruit development stages including 50 DAF (stage 1), 80 DAF (stage 2), 120 DAF (stage 3), 150 DAF (stage 4), 180 DAF (stage 5), and 220 DAF (stage 6). The *PME* genes were clustered according to their phylogenetic relationship. The color scale of heatmap represents the log_2_-transformed FPKM values. Dark red indicates high expression; light red indicates intermediate expression; and white indicates no expression

To further assess the spatial–temporal expression profiles of *CsPME* genes in navel orange fruits, the transcriptome data of 4 fruit tissues (namely, epicarp, albedo, segment membrane, and juice sac) at 6 development stages (namely, 0 DAF, 80 DAF, 120 DAF, 155 DAF, 180 DAF, and 220 DAF) of ‘Fengjie’ navel orange from our previous study were adopted (Supplementary Table S3) [41]. As shown in Fig. 4, of 53 *CsPME* family genes, 34 *CsPMEs* were expressed in navel orange fruits, indicating an indispensable role of *CsPME* genes in citrus fruits. *CsPME3*, *CsPME39*, *CsPME4*, and *CsPME52* were highly expressed in all the 4 fruit tissues. *CsPME20* and *CsPME36* were highly expressed in juice sacs and segment membranes. *CsPME6* and *CsPME16* were highly expressed in juice sacs. *CsPME26* and *CsPME22* were highly expressed in epicarps. Additionally, the expression levels of *CsPME3*, *CsPME4*, *CsPME20*, *CsPME21*, and *CsPME53* were decreased from 50 to 120 DAF, and then increased at 155 DAF, followed by a decrease until 220 DAF. The expression level of *CsPME39* was increased from 50 to 120 DAF, and then decreased from 155 to 180 DAF, followed by an increase at 220 DAF. The expression level of *CsPME16* was increased throughout the development stages, while *CsPME26* and *CsPME37* were decreased. In summary, these results indicated that *CsPME* genes might play significant roles in navel orange fruits development.

### Expression patterns of *CsPME* genes at different granulation levels of ‘lane late’ navel orange fruits

**Fig. 5:**
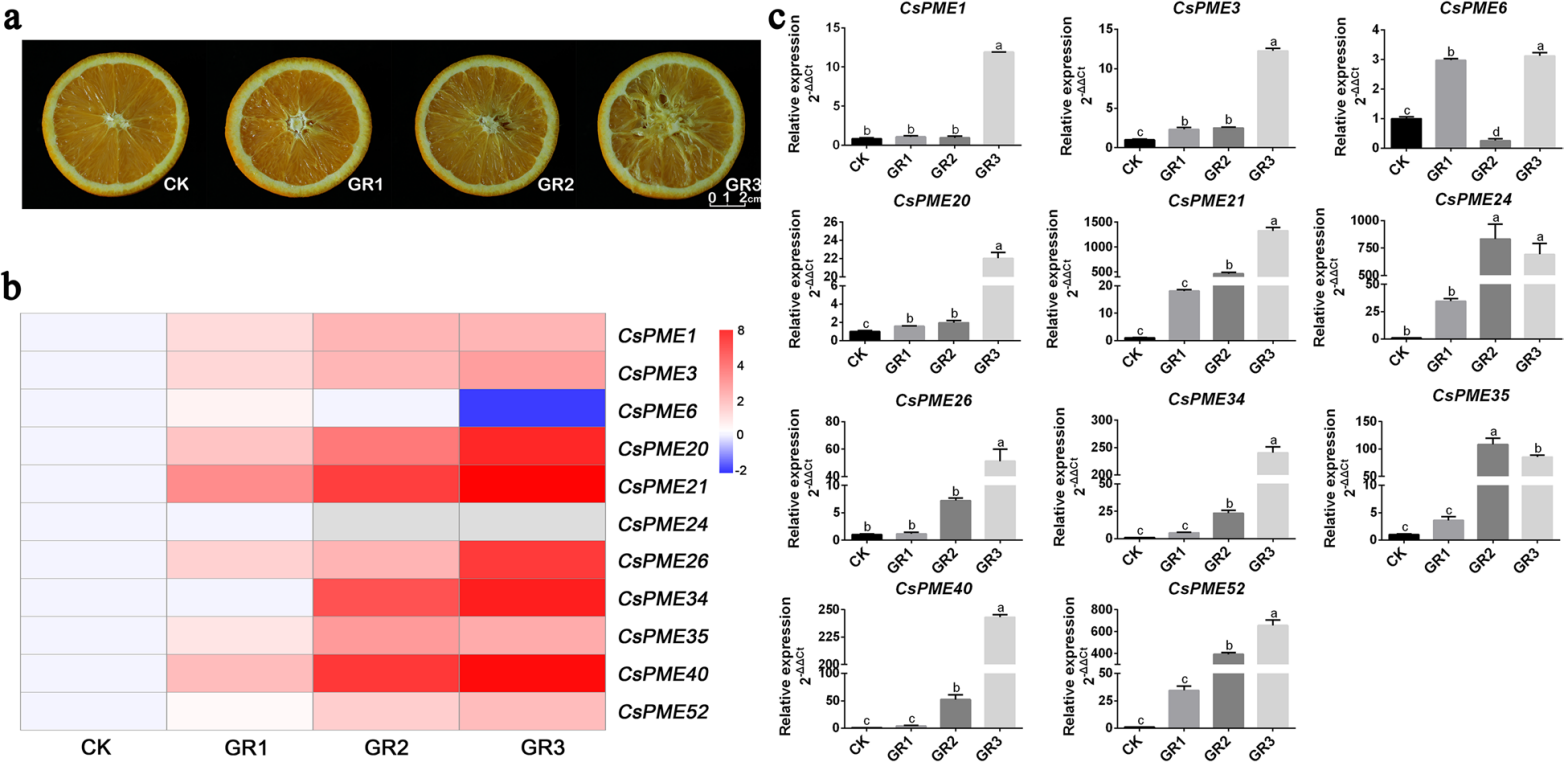
Expression patterns of *CsPMEs* in ‘Lane late’ navel range at different juice sac granulation levels. **a** ‘Lane late’ navel orange fruits of four juice sac granulation levels, including non-granulation (CK), slight granulation (GR1), moderate granulation (GR2), and serious granulation (GR3). **b** Differentially expressed *CsPMEs* at different juice sac granulation levels from transcriptome data. The color scale of heatmap represents the log_2_-transformed FPKM values. **c** qRT-PCR verification of the expression patterns of juice sac granulation-related *CsPMEs. CsEF1* and *β-actin* were used as internal controls, and three biological replicates were performed. All data were shown as means ± SE of three biological replicates. Bars with different lowercase letters indicated significant differences (*p* < 0.05 of one-way ANOVA in SPSS software)

To explore the functions of *CsPME* genes in juice sac granulation, we investigated the expression patterns of *CsPME* genes in ‘Lane late’ navel orange fruits at different granulation levels, including non-granulation (CK), slight granulation (GR1), moderate granulation (GR2), and serious granulation (GR3), by using transcriptome sequencing (Fig. 5a, Supplementary Table S4). As a result, 11 *CsPME* genes were differentially expressed in comparison of granulated samples and normal samples (Fig. 5b). Except for the down-regulation of *CsPME6*, other 10 differentially expressed *CsPME* genes were up-regulated in granulated fruit samples, indicating these *CsPMEs* might be involved in juice sac granulation of navel orange. Further, qRT-PCR was utilized to validate their expression profiles. As shown in Fig. 5c, most *CsPMEs* exhibited the same expression patterns as those in transcriptome data., but the expression pattern of *CsPME6* was opposite between transcriptome data and qRT-PCR at GR3 level. Most interestingly, *CsPME1*, *CsPME3*, and *CsPME6* were induced at the first granulation level (GR1), indicating these three *CsPMEs* might be involved in the initiation of juice sac granulation. In addition, we also assessed the expression patterns of other cell wall modification related genes during juice sac granulation of navel orange. As shown in Fig. S1, *CsPAL* (*phenylalanine ammonia lyase*), *CsCAD* (*cinnamyl alcohol dehydrogenase*), *CsPOD* (*peroxidase*), and *CsPG* (*polygalacturonase*) were induced in the granulated navel orange fruits, which further demonstrated that juice sac granulation progress was accompany with cell wall modifications.

### Expression patterns of *CsPME* genes under low temperature treatment

**Fig. 6:**
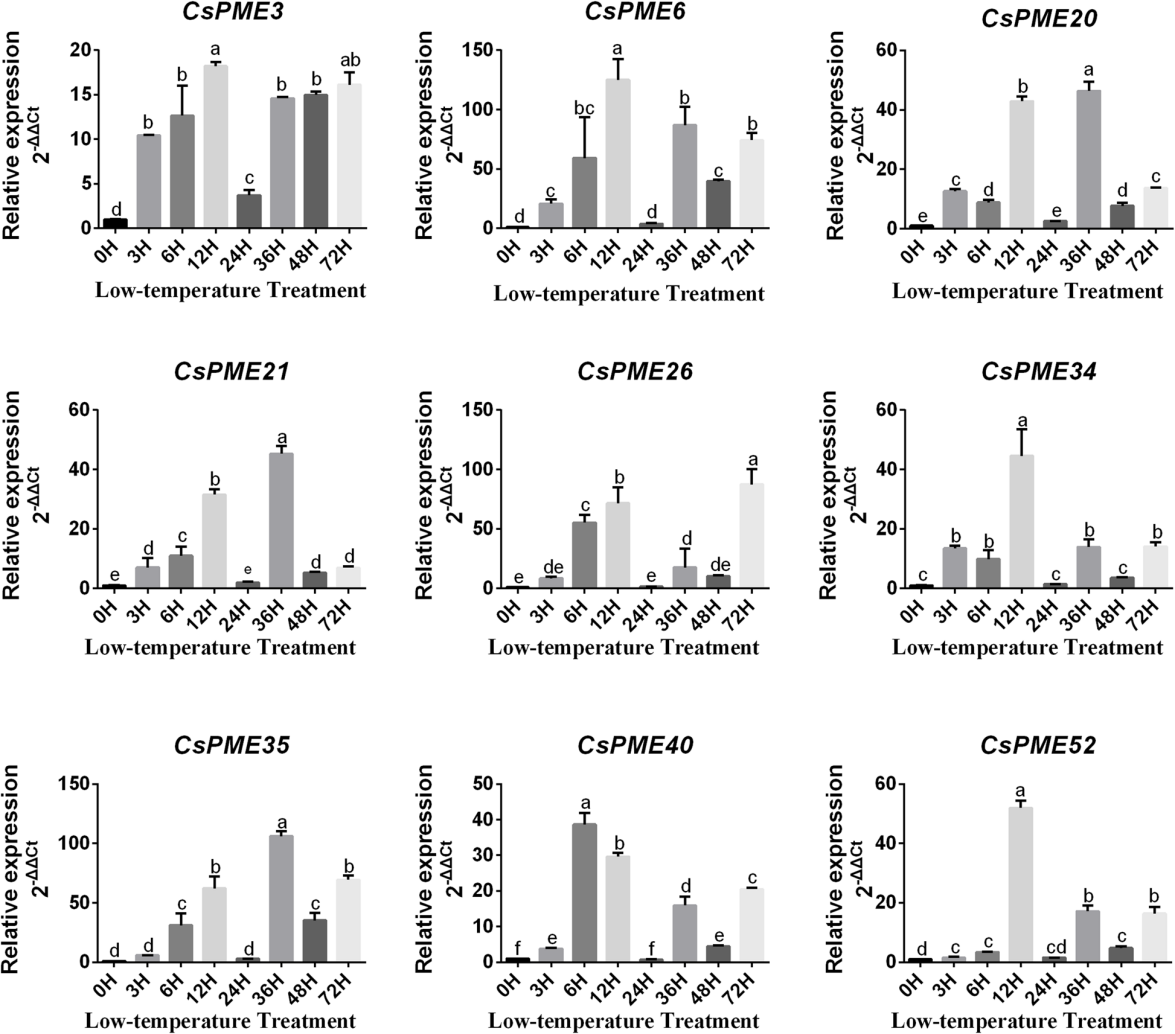
Expression profiles of juice sac granulation-related *CsPMEs* under low temperature treatment by using qRT-PCR. *CsEF1* and *β-actin* were used as internal controls, and three biological replicates were performed. All data were shown as means ± SE of three biological replicates. Bars with different lowercase letters indicated significant differences (*p* < 0.05 of one-way ANOVA in SPSS software

Our previous study had revealed that juice sac granulation of ‘Lane late’ navel orange in the Three Gorges area was mainly caused by the low temperature in the winter [37]. Thus, the expression patterns of *CsPMEs* under low temperature treatment were investigated in this study. As shown in Fig. 6, nine granulation-induced genes were also induced by low temperature treatment. Additionally*, CsPME3*, *CsPME20*, and *CsPME34* were induced at the beginning of treatment (3 h), indicating these three *CsPMEs* might be involved in the initiation of low temperature response in ‘Lane late’ navel orange. Notably, *CsPME3* was continuously highly-induced in all treatment group at all the detection time points, which further suggested a potential significant role of *CsPME3* in low temperature-induced juice sac granulation in ‘Lane late’ navel orange.

### Subcellular localization of *CsPME* genes

**Fig. 7:**
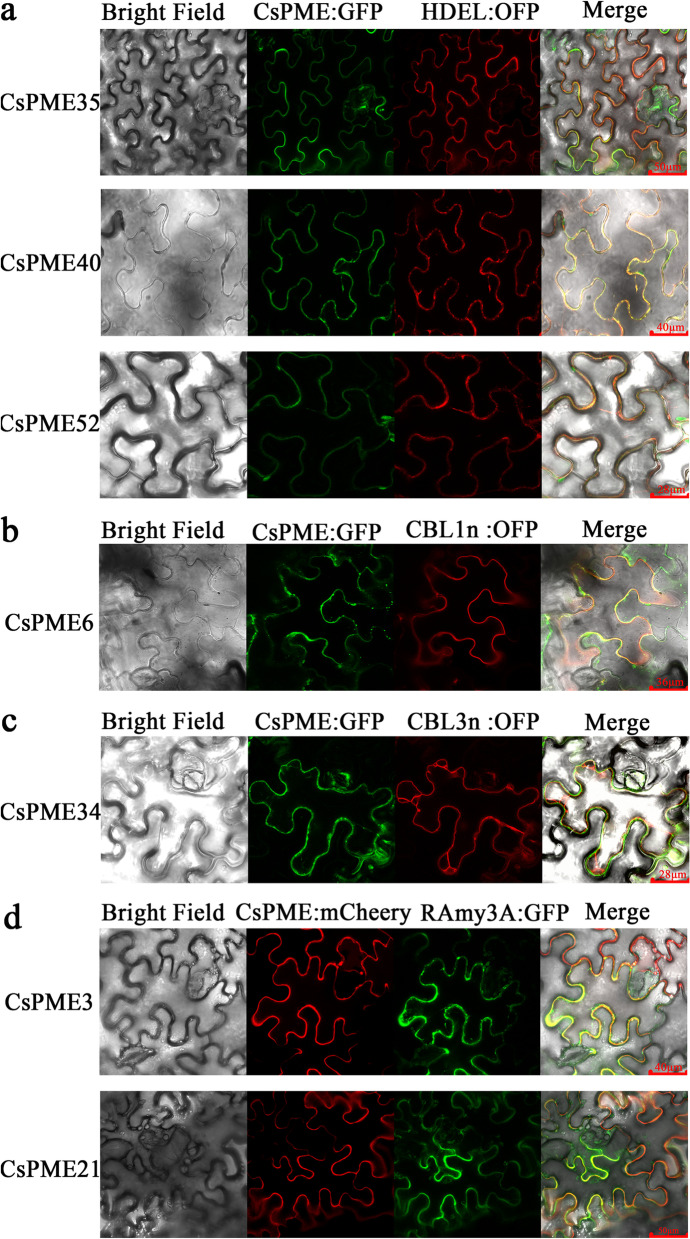
Subcellular localization of CsPME proteins in tobacco leaves. **a** The green fluorescence of CsPME35:GFP, CsPME40:GFP, and CsPME52:GFP was overlapped with the red fluorescence of endoplasmic reticulum marker (HDEL:OFP), respectively. **b** The green fluorescence of CsPME6:GFP was co-localized with the red fluorescence of plasma membrane marker (CBL1n:OFP). **c** The green fluorescence of CsPME34:GFP was co-localized with the red fluorescence of vacuole membrane marker (CBL3n:OFP). **d** The red fluorescence of CsPME3:mCherry and CsPME21:mCherry was co-localized with the green fluorescence of RAmy3A:GFP, respectively. *CsPME6*, *CsPME34*, *CsPME35*, *CsPME40*, and *CsPME52* were fused with GFP protein. *CsPME3* and *CsPME21* were fused with mCherry protein. ER-marker (HDEL:OFP), apoplast marker (RAmy3A:GFP), plasma membrane marker (CBL1n:OFP), and vacuole membrane marker (CBL3n:OFP) were utilized for co-localization analysis and imaging

To explore the subcellular localization of CsPME proteins, the coding sequence (CDS) of *CsPMEs* (including *CsPME6*, *CsPME34*, *CsPME35*, *CsPME40*, and *CsPME52*) without stop codon was ligated with that of green fluorescent protein (GFP) and cloned into pICH86988 vectors, and these vectors were co-expressed with corresponding markers in tobacco leaves. As shown in Fig. 7a, the green fluorescence of CsPME35:GFP, CsPME40:GFP, and CsPME52:GFP were overlapped with the red fluorescence of endoplasmic reticulum marker (HDEL:OFP), revealing that CsPME35, CsPME40, and CsPME52 were localized in the endoplasmic reticulum. Additionally, the green fluorescence of CsPME6:GFP, and CsPME34:GFP were co-localized with the red fluorescence of plasma membrane marker (CBL1n:OFP) and vacuole membrane marker (CBL3n:OFP), respectively (Fig. 7b, c).

According to a previous study, the weaker green fluorescence in the apoplast might be caused by the lower stability of the protein in the apoplast than that in the endoplasmic reticulum [42]. Thus, the CDS of *CsPME3* and *CsPME21* was further ligated with that of mCherry and cloned into pICH86988 vectors, and these vectors were co-expressed with an apoplast marker RAmy3A:GFP. As shown in Fig. 7d, the red fluorescence of CsPME3:mCherry and CsPME21:mCherry was respectively co-localized with the green fluorescence of RAmy3A:GFP, indicating that CsPME3 and CsPME21 were localized in the cell wall. Based on these, we hypothesized that *CsPME3* and *CsPME21* might function as pectin-degrading enzymes in cell wall, and they might be involved in the low temperature-induced juice sac granulation of navel orange.

### Identification of interactive transcription factor of pro*CsPME3*

**Fig. 8:**
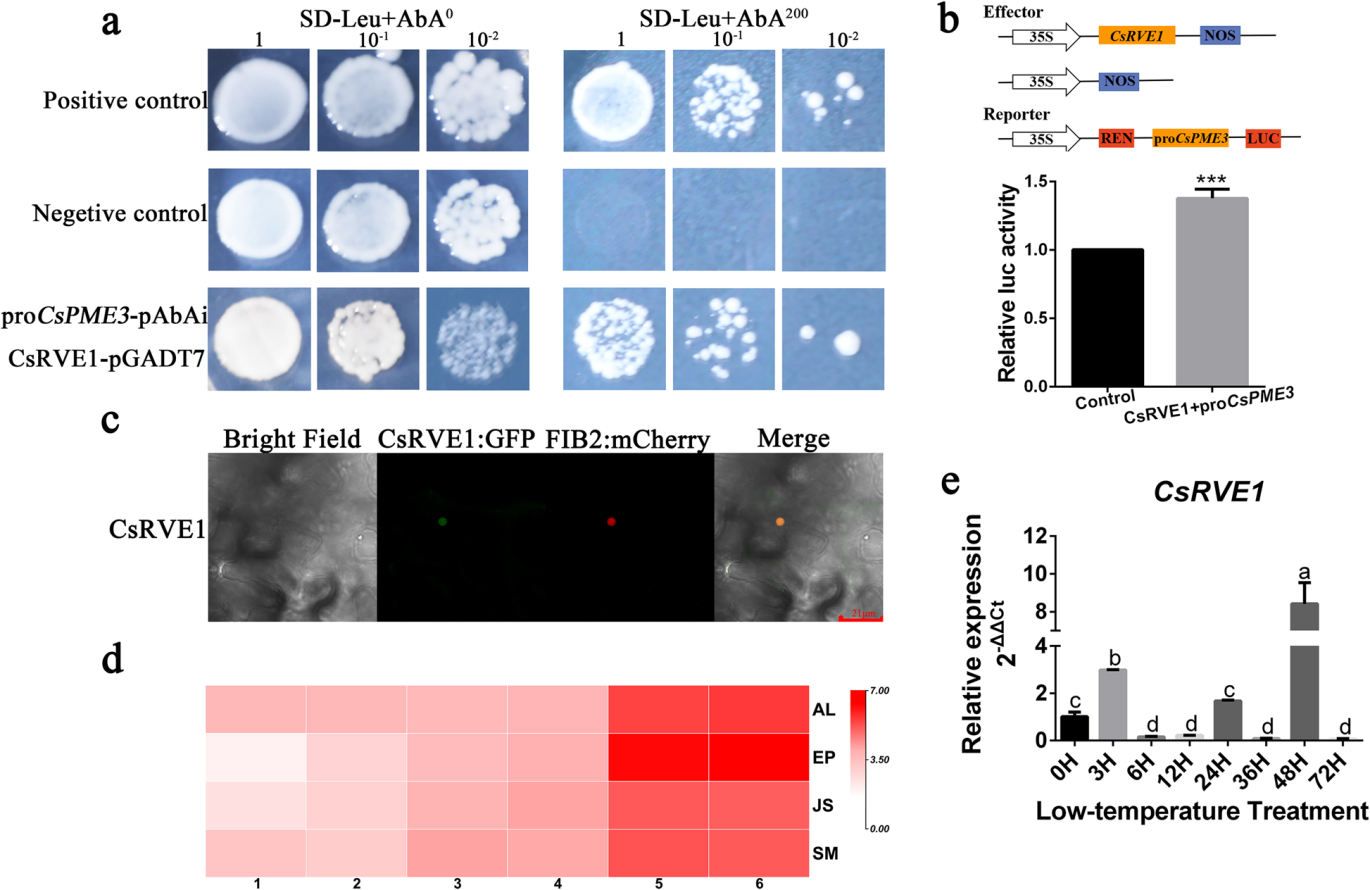
Transcription factor *CsRVE1* bound to the promoter of *CsPME3* and activated its activity. **a** Yeast one-hybrid assay suggested the interaction between *CsRVE1* and the promoter of *CsPME3*. **b** Dual luciferase transcriptional activity assay revealed the activation of pro*CsPME3 by CsRVE1.* **c** Subcellular localization of *CsRVE1* in tobacco leaves. *CsRVE1* was fused with GFP, and nucleolus marker (FIB2:mCherry) was utilized for co-localization analysis and imaging. **d**. Spatial and temporal expression profiles of *CsRVE1* in navel orange fruits. The color scale of heatmap represents the log_2_-transformed FPKM values. **e**. Expression patterns of *CsRVE1* under low temperature stress. *CsEF1* was used as internal control, and three biological replicates were performed. Three individual biological replicates were performed for Dual luciferase transcriptional activity assay and qRT-PCR, data were shown as means ± SE of three biological replicates, and bars with different lowercase letters indicated significant differences (*p* < 0.05 of one-way ANOVA in SPSS software)

Due to the important role of *CsPME3* in low temperature-induced juice sac granulation, we further explored the regulation network of *CsPME3*. The 2 kb upstream sequence of *CsPME3* was cloned as promoter sequence, and the *cis*-elements of pro*CsPME3* was analyzed using PlantCARE online tool. As shown in Fig. S2, *cis*-elements related to plant hormones (ABRE, TGACG-motif, CGTCA-motif, TCA-element, and TGA-element) and biotic/abiotic stress response (TC-rich repeats) were detected in the promoter of *CsPME3*. Interestingly, MYB recognition site was also found in the pro*CsPME3*, indicating that *CsPME3* might be regulated by MYB transcription factors. To further identify the regulatory transcription factor of *CsPME3*, the promoter sequence of *CsPME3* was ligated into pAbAi to construct bait vector. Then, the bait vector was utilized to screen the yeast one-hybrid (Y1H) library, and 8 positive clones were obtained. The point-to-point Y1H assay indicated that of 8 positive clones, only the clones of *CsRVE1* (Cs6g16000) + pro*CsPME3* were grown well on the SD-leu + 200 ng/µL AbAi (aureobasidin A) medium (Fig. 8a), indicating that *CsRVE1* directly interacted with the promoter of *CsPME3*. Dual luciferase transcriptional activity assay (LUC) was further performed on tobacco leaves to investigate the regulation effect of *CsRVE1* on *CsPME3* with *CsRVE1* as effector and pro*CsPME3* as reporter. As shown in Fig. 8b, the activity of pro*CsPME3* was significantly induced by *CsREV1*. In summary, these results suggested that the transcription factor *CsREV1* was involved in low temperature-induced juice sac granulation by regulating the activity of *CsPME3*.

### Subcellular localization and expression patterns of *CsRVE1*

To investigate the subcellular localization of *CsRVE1*, coding sequence of *CsRVE1* was fused with GFP, cloned into pICH86988 vector, and co-expressed with nucleolus marker [43]. As shown in Fig. 8c, the green fluorescence of CsRVE1:GFP was co-localized with the red fluorescence of nucleolus marker (FIB2:mCherry), suggesting that CsRVE1 protein was localized to the nucleolus. Further, the spatial and temporal expression pattern of *CsRVE1* during fruit development was evaluated based on our previous transcriptome data of 4 fruit tissues during 6 development stages of navel orange. As shown in Fig. 8d, *CsRVE1* was highly expressed in all the 4 fruit tissues, which was in line with the expression pattern of *CsPME3*. In addition, the expression level of *CsRVE1* was continuously increased throughout the 6 fruit development stages, indicating that *CsRVE1* might be involved in the fruit ripening process. Moreover, we also found that the expression level of *CsRVE1* was induced by low temperature treatment at 3 h and 48 h (Fig. 8e). Taken together, these results further implied that *CsRVE1* might play an important regulatory role in low temperature-induced juice sac granulation.

## Discussion

The pectin methylesterases (PME) family plays a crucial role in fruit ripening and softening, pollen development and growth, root hair formation, seed gemination, biotic and abiotic stress response, and other developmental processes in plants [20–26]. According to its significant functions, the *PME* gene family was widely identified in various plant species. However, the classification and biological functions of *PME* gene family remains largely unclear in citrus. In this study, a total of 53 *CsPME* genes were identified from citrus via genome-wide analysis. The number of *PME* genes was larger in citrus than in *O. sativa* (43) [17], but smaller than in *A. thaliana* (66) [14], *G. arboretum* (80) [16], *B. rapa* (110) [15], and *L. usitatissimum* (105) [18]. The phylogenetic analysis revealed that *CsPME* genes were clustered into four clades, which was further verified by the gene structure and conserved motifs analyses. The gene duplication analysis revealed that segmental duplication events might contribute more to the expansion of *CsPME* genes than tandem duplication events.

The spatial and temporal expression profiles provide important clues for investigating the biological function of *CsPME* genes. The high-spatiotemporal-resolution transcriptome data of navel orange fruits from our previous study [41] indicated that much more *CsPME* family genes (around 64%, 34 out of 53) were expressed in citrus than in tomato (27 *SlPMEs*) and strawberry (5 *FvPMEs*) during fruit development [19]. These results strongly suggested that *CsPMEs* might play indispensable roles in fruit development in navel orange. Some *CsPME* genes exhibited tissue-specific expression patterns. For example, *CsPME20* and *CsPME36* were highly expressed in juice sac and segment membrane, *CsPME6* and *CsPME16* in juice sac, and *CsPME26* and *CsPME22* in epicarp, suggesting that these *CsPME* genes might have particular function in the development of specific tissues. However, *CsPME3*, *CsPME39*, *CsPME4*, and *CsPME52* were highly expressed in all the fruit tissues. The phylogenetic analysis demonstrated that *CsPME3* was clustered together with *AtPME3* (At3g14310, designed as *AtPME29* in Fig. 1) which was particularly active in leaves, stems, and roots of Arabidopsis seedlings [44]. Moreover, *AtPME3* was reported to play significant roles in adventitious rooting, root hypersensitivity to zinc, plant-nematode interaction, pathogen infection, and seed gemination [25, 44–47]. Thus, *CsPME3*, *CsPME39*, *CsPME4*, and *CsPME52* might have multiple biological functions in fruits development. Additionally, the expression levels of those highly expressed *CsPME* genes, including *CsPME3*, *CsPME4*, *CsPME20*, *CsPME21*, and *CsPME53*, were decreased from 50 to 120 DAF, and then increased at 155 DAF, followed by a decrease until 220 DAF. The 155 DAF was the late fruit expansion stage, and 220 DAF was the fruit ripening stage [41], indicating these genes might be involved in the fruit expansion and ripening in navel orange.

Most interestingly, 11 *CsPME* genes were found to be differentially expressed in comparison of granulated fruits and non-granulated fruits in both transcriptome data and qRT-PCR results of navel orange, indicating that these *CsPME* genes might be related to the juice sac granulation of ‘Lane late’ navel orange. Additionally, 10 *CsPME* genes were induced in the granulated fruits, which was consistent with our previous study of navel orange [37], but disagreed with the findings in Ponkan mandarin [39]. We deduced that this variation could be attributed to the different cell wall metabolism between tight skin and loose skin varieties of citrus [48]. Moreover, *CsPME1*, *CsPME3*, and *CsPME6* were induced at the first granulation level (GR1) with less than 25% granulation area of total fruit (Fig. 5b), indicating that these genes might be involved in the initiation of juice sac granulation. Our previous study reported that the juice sac granulation of ‘Lane late’ navel orange was mainly due to the low temperature in the winter [37], and this study found that 9 juice sac granulation- related *CsPME* genes were induced by low temperature treatment, especially at 6 h, which was in line with previous studies results that *PME* family genes were up-regulated by cold stress in *B.napus* and *A. thaliana* [49–51]. It should be noted that *CsPME3* was consecutively highly-expressed at all the detection time points, which further suggested that *CsPME3* might be involved in the initiation of low temperature-induced juice sac granulation in ‘Lane late’ navel orange.

The subcellular localization analysis revealed that three CsPME proteins were localized on the endoplasmic reticulum, one on plasma membrane, one on vacuole membrane and two on the apoplast, which strongly supported the hypothesis that *CsPME* genes played multiple biological functions in citrus fruits. Most interestingly, *CsPME3* and *CsPME21* were localized on the apoplast (cell wall), which was in accordance with the findings of its orthologous genes, *AtPME3* and *FvPME39,* in Arabidopsis and strawberry, respectively [19, 44]. Additionally, *AtPME3* was ubiquitously expressed in *A. thaliana*, particularly in vascular tissues, and *AtPME3* was functioned as a pectin-degrading enzyme during the cell wall remodeling process [25, 44, 52]. *FvPME39* was identified as pectin-modifying enzyme to regulate fruit firmness, pectin content, and cell wall structure during fruit softening [19]. The above-mentioned findings indicated that *CsPME3,* as a pectin-modifying enzyme, might play significant roles in cell wall structure modification and fruit rigidification during juice sac granulation in ‘Lane late’ navel orange.

**Fig. 9:**
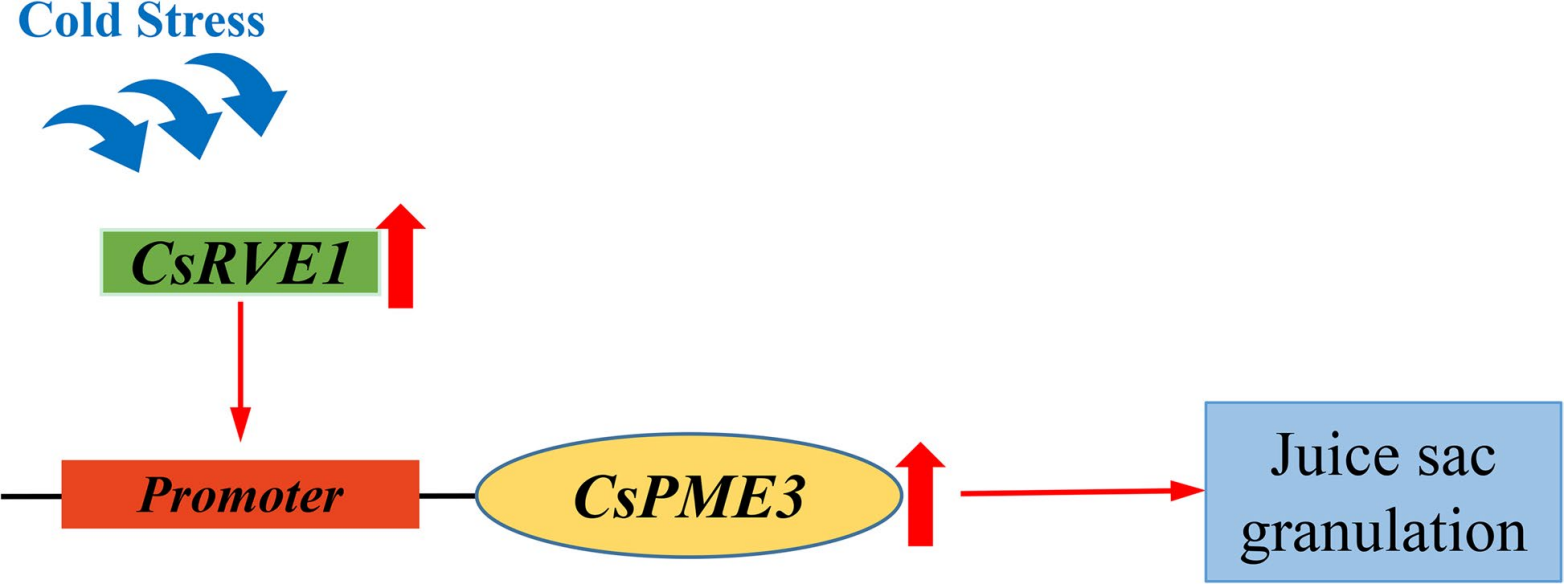
Schematic diagram showing the cold-induced regulation pattern *CsRVE1* on pro*CsPME3* involved in juice sac granulation of navel orange. The cold weather in the deep winter induces the expression of transcription factor *CsRVE1*, then *CsRVE1* binds to the promoter of *CsPME3* and thus activates the expression of *CsPME3*, which further leads to the juice sac granulation of ‘Lane late’ navel orange fruits

To further reveal the regulation mechanism of *CsPME3* during juice sac granulation, the promoter sequence of *CsPME3* was utilized as a bait to screen a yeast one-hybrid library. The transcription factor *CsRVE1* was found to directly interact with the pro*CsPME3*. Additionally, LUC assay further illustrated that *CsRVE1* activated the activity of pro*CsPME3,* and the subcellular localization analysis showed that *CsRVE1* was localized to the nucleolus. These results suggested that *CsRVE1* was nucleolus-localized transcription activator of pro*CsPME3*. *CsRVE1* (*REVEILLE1*) was an ortholog of *AtRVE1* and belonged to a subfamily of Myb-like transcription factors that includes *CIRCADIAN CLOCK-ASSOCIATED 1* (*CCA1*) and *LATE ELONGATED HYPOCOTYL* (*LHY*) clock components [53, 54]. *RVE1* was reported to regulate the auxin biosynthesis, seed dormancy and germination, chlorophyll biosynthesis during plant development [53, 55, 56]. *AtRVE1* acted as a negative regulator of freezing tolerance in Arabidopsis [57]. Here, we also found that the expression level of *CsRVE1* was induced by low temperature stress. Thus, it could be concluded that *CsRVE1* might be a low temperature-induced nucleolus-localized transcription activator of *CsPME3.* The cold weather in the deep winter induced the expression of transcription factor *CsRVE1*, then *CsRVE1* binds to the promoter of *CsPME3* and thus activated the expression of *CsPME3.* Subsequently, the activated pectin methylesterase activity leads to the modification of cell wall components, and thus resulted in the juice sac granulation of ‘Lane late’ navel orange fruits (Fig. 9).

## Conclusions

In summary, a total of 53 *CsPME* genes were identified from *Citrus sinensis*, including 29 Type-I and 25 Type-II *CsPMEs.* Comprehensive bioinformatics analyses of phylogenetic relationship, gene structure, conserved domains, chromosome localization, gene duplication, and synteny were performed on *CsPME* genes, which provide important clues for further investigation of *CsPME* genes. The expression profiles of *CsPMEs* and their subcellular localization analysis revealed that the *CsPME g*enes were involved in the low temperature-induced juice sac granulation in navel orange fruits. Moreover, the Y1H and LUC assays revealed the regulatory effect of *CsRVE1* on the activity of pro*CsPME3*. Our results provide an insight into evolution, expression profiles, subcellular localization, and regulation patterns of the *PME* gene family in citrus. This study lays a solid foundation for further identification of the biological functions of *CsPME* gene during juice sac granulation of citrus.

## Methods

### Identification of putative *PME* family genes from* Citrus sinensis*

To identify *CsPME* genes, genome resources of sweet orange (*Citrus sinensis*, version 2) were downloaded from the *Citrus Sinensis* Annotation Project of Huazhong Agricultural University (http://citrus.hzau.edu.cn/orange/) [40], and two BLAST methods were conducted. Firstly, the amino acid sequences of HMM (Hidden Markov Model, http://hmmer.janelia.org/) profile of the pectin Methyl-esterase (PME) domain (PF01095) were downloaded from Pfam database (http://pfam.xfam.org/, version 33.1) [58], and then used as queries for retrieving candidate *PME* family genes in *C. sinensis* genome using HMMER software (version 3.0, http://hmmer.org/) with an E-value cutoff of 1e-3 [59]. Secondly, the protein sequences of all known *PME* family genes in *Arabidopsis thaliana* were downloaded from TAIR database (http://www.Arabidopsis.org/), and then utilized as queries for retrieving candidate *PME* family genes in *C. sinensis* genome by using BLAST-P with E-value cutoff of 1e-5. To ensure the presence of *PME* domain for each protein, all obtained candidate *PME* family genes were further confirmed by Simple Modular Architecture Research Tool (SMART, http://smart.embl-heidelberg.de/) and Conserved Domain Database (CCD, http://www.ncbi.nlm.nih.gov/cdd/) [60, 61]. Finally, the non-redundant and confirmed candidate genes were considered as citrus *PME* family genes, and the physicochemical properties of all CsPME proteins were predicted using ExPASy tool (http://web.expasy.org/protparam/) [62]. The gene loci of *PME* family genes were downloaded from *Citrus Sinensis* Annotation Project of Huazhong Agricultural University [40], and visualized using TBtools software [63]. The cis-elements of the promoter sequence of *CsPME3* was analyzed using PlantCARE online tool (http://bioinformatics.psb.ugent.be/webtools/plantcare/html/) according to the default parameters [64].

### Phylogenetic analysis, gene structure, and conserved motifs of *PME* family genes

To explore the phylogenetic relationship of *PME* family genes between *C. sinensis* and *A. thaliana*, a multiple sequence alignment was performed using MAFFT (Multiple Alignment using Fast Fourier Transform, https://mafft.cbrc.jp/alignment/server/) with default parameters [65], and a phylogenetic tree was subsequently constructed using Maximum Likelihood Method of MEGA software with 1000 bootstraps based on the protein sequences of PMEs from *C. sinensis* and *A.thaliana*. [66]. The final phylogenetic tree was visualized and polished with the Interactive Tree of Life (iTOL, version 5, https://itol.embl.de/) [67]. The protein sequences of *A. thaliana* were downloaded from TAIR database (http://www.Arabidopsis.org/). The gene structures (exon–intron organizations) of *PME* family genes were presented using TBtools software. The conserved motifs were detected through the online tool of Multiple Em for Motif Elicitation program (MEME, version 5.1.1, http://meme-suite.org/tools/meme) using classic mode with the following parameters: (1) zero or one occurrence per sequence (zoops), (2) the optimum motif width ranging from 6 to 50, (3) 15 as the maximum motif number [68, 69]. Subsequently, the identified motifs were further annotated against an online database of InterPro (version 80.0, www.ebi.ac.uk/interpro/search/sequence/) [70]. Finally, the combination figure of the phylogenetic tree, gene structures, and conserved motifs of *PME* family genes was generated using TBtools software [63].

### Gene duplication and synteny analysis

The gene duplication of *CsPME* family genes was determined according to the criteria reported previously [71]. Two genes separated by less than 5 genes within 100 kb chromosome fragment were considered as tandemly duplicated genes [72]. The segmentally duplicated genes of *CsPME* family genes were identified with Multiple Collinearity Scan toolkit (MCScanX, [73]), and Circos software of TBtools was utilized for visualization [63]. MCScanX was further employed to analyze synteny relationship of the ortholog of *PME* family genes from *C. sinensis*, *A. thaliana*, and *O. sativa* with the default parameters. Synteny maps were generated by Dual Systeny Plotter software based on TBtools [63].

### Plant materials and treatments

All plant materials used in this study were collected from mature ‘Lane late’ navel orange (*Citrus sinensis* Osbeck) trees in Zigui County of Hubei Province in the three gorgers reservoir area (E 110°41’, N 30°54’). Granulated ‘Lane late’ fruit samples were harvested at 350 DAF (days after flowering) in 2018 due to the extreme cold weather in the winter in reference to our previous study [37]. The average temperature in January of sampling site was -4 ℃ with an altitude of 520 m, and the soil property was red yellow sandy soil with a pH value of 6.3. Each Fruit was halved by sterilized knife, and then divided into four granulation levels, namely, non-granulation (CK), slight granulation (GR1), moderate granulation (GR2), and serious granulation (GR3), according to a previous study [74]. Finally, the fruits samples at different granulation levels were collected. For low temperature treatment, the normal ‘Lane late’ fruits were placed on a plate and covered with sterilized gauze to keep moist. Then, the fruits were placed in an incubator at 4 ℃. The samples were collected at 0 h, 3 h, 6 h, 12 h, 24 h, 48 h, and 72 h. Each sample had three biological replicates with 3 individual fruits per biological replicate. All fruit samples were immediately frozen with liquid nitrogen and stored in –80 ℃ for RNA extraction.

### Expression profiles of *PME* family genes in *C. sinensis*

The transcriptome data of 4 fruit tissues (including epicarp, albedo, segment membrane, and juice sac) at 6 development stages (including 50 DAF, 80 DAF, 120 DAF, 155 DAF, 180 DAF, and 220 DAF) of ‘Fengjie’ navel orange from our previous study were utilized to investigate the spatial and temporal expression patterns of *CsPME* genes [41]. Specifically, the fruits were sampled in the second physiological fruit-falling period (50 day after flowering, DAF), in the expansion period (80, 120, and 155 DAF), in the coloring period (180 DAF), and in the full-ripening period (220 DAF). The transcriptional expression patterns of three independent bio-replicates were calculated by Fragments Per Kilobase per Million (FPKM). The heatmap was drawn using heatmap package of TBtools based on the log_2_-transformed FPKM values [63].

To identify the granulation-related *CsPME* genes, a transcriptome analysis was performed in ‘Lane late’ navel orange fruits at 4 different granulation levels, including non-granulation (CK), slight granulation (GR1), moderate granulation (GR2), and serious granulation (GR3). Total RNA was extracted from 12 ‘Lunwan’ navel orange fruit samples (three biological replicates) using TRIzol™ reagent according to the manufacturer’s instructions (Thermo Scientific). RNA quality was assessed by agarose gel electrophoresis and Agilent 2100 Bioanalyzer (Agilent Technologies), and then qualified RNA of all 12 samples were subjected to Berry Genomics (Beijing) for library construction and transcriptome sequencing. The gene expression level was assessed using HTSeq with FPKM method [75]. The differential expression analysis between pair of samples was performed using edgeR package [76], and genes with |Log_2_ fold change|≥ 1 and p-adjusted value ≤ 0.05 were considered as differentially expressed genes. Heatmap of differentially expressed *CsPME* genes was performed using R package based on the Log_2_ fold change values.

### RNA extraction, cDNA synthesis, and qRT-PCR of *PME* family genes in* C. sinensis*

Total RNA was isolated from different samples using TRIzol® Reagent (Invitrogen, USA) according to the manufacturer’s instructions. RNA quality was assessed by agarose gel electrophoresis, and then one microgram of total RNA was used for ss/dsDNA digestion and cDNA synthesis with a 5X All-In-One RT MasterMix with AccuRT (Applied Biological Materials). Quantitative RT-PCR was performed on 7500 Fast Real-Time PCR System (Applied Biosystems) with an EvaGreen 2X qPCR MasterMix (Applied Biological Materials). *Elongation factor 1* (*Ef1*, Cs8g16990.1) and *β-actin* (Cs1g05000.1) were selected as internal references to reduce the systematic variance, and the relative abundance of *PME* genes was calculated with the average Ct values of *Ef1* and *β-actin* using 2^−ΔΔCt^ method [77]. The primers used for qRT-PCR were listed in Supplementary Table S5.

### Subcellular localization analysis

The coding sequence (CDS) of *PME* family genes (*PME4*, *PME8*, *PME30*, *PME51*, *PME54*, *PME55*, *PME76*, and *PME95*) without stop codon was cloned from ‘Lane late’ navel orange fruit cDNA, ligated with that of GFP module or mCherry module (exclusively for the genes localized on the apoplast), and cloned into pICH86988 vectors to construct 35S::*PMEs*:*GFP*, and 35S::*PMEs*:*mCherry* vectors through Golden Gate method [78]. The resultant vectors were transformed into *Agrobacterium tumefaciens* (strain GV3101 with pSoup-p19). The 35S::*PMEs*:*GFP* and 35S::*PMEs*:*mCherry* vectors together with corresponding markers were then co-expressed in tobacco leaves, including ER-marker (HDEL:OFP) [79], plasma membrane marker (CBL1n:OFP) [79], vacuole membrane marker (CBL3n:OFP) [79], and apoplast marker (RAmy3A:GFP) [80]. Additionally, the 35S::*CsRVE1:GFP* vector was constructed as described above and co-expressed with nucleolus marker (FIB2:mCherry) [43]. Transient expression experiment was performed by a previously-reported method [81]. The transformed tobacco plants were cultured in green house for 3 days, then the subcellular localization of *PME* family genes was observed and photographed via a confocal laser-scanning microscope (TCS SP6, Leica). The primers used for vector construction were listed in Supplementary Table S5.

### Yeast one-hybrid assay

Yeast one-hybrid assay was conducted using the method reported by Lu et al. [82]. Specifically, the promoter sequence of *PME3* was amplified from the genomic DNA extracted from ‘Lane late’ navel orange leaf. The promoter sequence was cloned into pAbAi vector to construct *PME3-*pAbAi bait vector. Subsequently, the obtained bait vector was transformed into Y1H Gold yeast, and the positive clones were selected with synthetic dextrose media lacking uracil (SD-Leu). Then, the positive bait was used to screen interactive transcription factors from a citrus Y1H library provided by Jihong Liu lab of Huazhong agricultural university. The pGADT7 empty vector (as a negative control) was transformed into yeast competent cell containing bait. The transformed yeast cells were diluted with a 10 × dilution series and dotted onto SD-Leu plates with or without aureobasidin A (AbAi). If the cells grew on both types of media with or without AbAi, the interaction of prey proteins and bait sequences was identified. The primers used for constructing Y1H vectors were listed in Supplementary Table S5.

### Dual luciferase transcriptional activity assay

The dual luciferase transcriptional activity (LUC) assay was performed according to a previously reported method [82]. To be more specific, the promoter sequence of *PME3* was inserted into the upstream of LUC coding sequence in pGreen 0800-LUC to construct promoter-LUC reporter vector. The CDS of *CsRVE1* was cloned into AML4 over-expression vector to construct 35S::*CsRVE1* effector vector. The effector and reporter vectors were transformed into *Agrobacterium tumefaciens* (strain GV3101 with pSoup-p19), and then co-expressed in tobacco leaves at an effector: reporter ratio of 1: 1 according to the method described by Hellens et al. [83]. After 3-day cultivation in green house, the LUC activity was determined using the Dual-Luciferase Reporter Assay System (Promega) with an infinite200 Pro microplate reader (Tecan). The primers used for constructing LUC vectors were listed in Supplementary Table S5.

## Supplementary Information

**Additional file 1: Figure S1.** The expression patterns of four cell wall modification genes during juice sac granulation of navel orange.

**Additional file 2: Figure S2.** The predicted* cis*-elements of the promoter sequence of* CsPME3*.

**Additional file 3: Table S1.** Verification of PME and PMEI domains in candidate* CsPME* genes.

**Additional file 4: Table S2.** Basic information of the *PME* genes in *Citrus sinensis*.

**Additional file 5: Table S3.** The expression levels of *CsPMEs* in the transcriptome data of 4 fruit tissues at 6 development stages in ‘Fengjie' navel orange. Four fruit tissues including albedo (AL), epicarp (EP), juice sac (JS), and segment membrane (SM), and six fruit development stages including 50 DAF (stage 1), 80 DAF (stage 2), 120 DAF (stage 3), 150 DAF (stage 4), 180 DAF (stage 5), and 220 DAF (stage 6).

**Additional file 6: Table S4.** The expression levels of* CsPMEs* in the transcriptome data of ‘Lane late’ navel orange fruits under 4 different granulation levels, including non-granulation (CK), slight granulation (GR1), moderate granulation (GR2), and serious granulation (GR3).

**Additional file 7: Table S5.** List of primers used for qRT-PCR, gene cloning, and vector construction in this study.

## Data Availability

All Arabidopsis protein sequences were downloaded from The Arabidopsis Information Resource (TAIR) (https:// www.arabidopsis.org), all Citrus protein sequences were downloaded from the *Citrus Sinensis* Annotation Project (http://citrus.hzau.edu.cn/orange/). The transcriptome data used in this study can be accessed with the Gene Expression Omnibus (GSE125726) from the NCBI database. The amplified coding sequences of *CsPME3*, *CsPME6*, *CsPME21*, *CsPME34*, *CsPME35*, *CsPME40*, *CsPME52*, and *CsRVE1* can be accessed in the International Nucleotide Sequence Database Collaboration (INSDC) with the accession number of OM100720 ~ OM100727, and the amplified promoter sequence of *CsPME3* can be accessed with the accession number of OM100728 in INSDC.

## Abbreviations

PME: Pectin methylesterase
Chr: Chromosome
CDS: Coding sequence
FPKM: Fragments per kilobase of transcript per million mapped reads
Y1H: Yeast one-hybrid assay
LUC: Dual luciferase transcriptional activity assay
GFP: Green fluorescent protein
OFP: Orange fluorescent protein
mCherry: mCherry red fluorescent protein
AbAi: Aureobasidin A
