# Supplementary figures and images for: Characterization of pectin methylesterase gene family and its possible role in juice sac granulation in navel orange (Citrus sinensis Osbeck)
Source: BMC Genomics. 2022 Mar 7;23:185. doi: 10.1186/s12864-022-08411-0 (PMC8900419; doi:10.1186/s12864-022-08411-0)

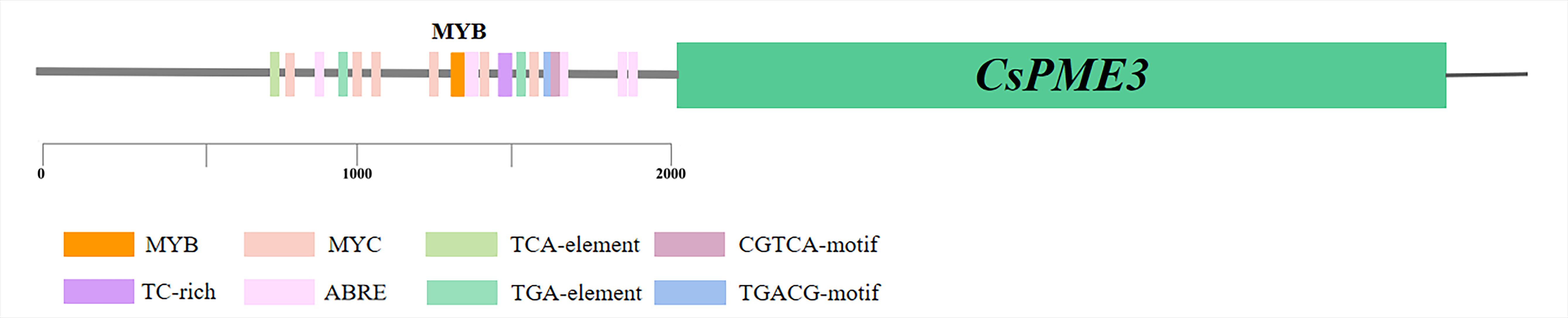

Supplement: Supplementary file 2 — Additional file 2: Figure S2. The predicted cis-elements of the promoter sequence of CsPME3. [file 12864_2022_8411_MOESM2_ESM.tif]
